# Clinical Features Associated With Malignant Transformation of Low‐Grade Dysplasia

**DOI:** 10.1111/jop.70070

**Published:** 2025-10-06

**Authors:** Denise M. Laronde, Matt Berkowitz, A. Ross Kerr, Erinn M. Hade, Mutita Siriruchatanon, Miriam P. Rosin, Stella K. Kang

**Affiliations:** ^1^ Oral Biological and Medical Science, Faculty of Dentistry University of British Columbia Vancouver Canada; ^2^ Cancer Control Research British Columbia Cancer Research Institute Vancouver Canada; ^3^ Statistics and Actuarial Science Simon Fraser University Burnaby Canada; ^4^ Department of Oral and Maxillofacial Pathology, Radiology, and Medicine New York University College of Dentistry New York New York USA; ^5^ Department of Population Health New York University Grossman School of Medicine New York New York USA; ^6^ Department of Radiology New York University Grossman School of Medicine New York New York USA; ^7^ Department of Biomedical Physiology and Kinesiology Simon Fraser University Burnaby Canada

**Keywords:** malignant transformation, oral epithelial dysplasia, oral squamous cell carcinoma, toluidine blue

## Abstract

**Background:**

Inferring risk for malignant transformation (MT) in patients with lesions diagnosed as mild or moderate oral epithelial dysplasia (low‐grade OED) remains challenging. We developed two models assessing the risk of progression to high‐grade OED (severe dysplasia or carcinoma in situ) or OSCC in patients with low‐grade OED lesions.

**Methods:**

We included demographic, risk habit and clinical data from participants with low‐grade OED lesions enrolled in the BC Oral Cancer Prevention Program's Oral Cancer Prediction Longitudinal study. Cox proportional hazard models were fit to estimate the effects of anatomic site and toluidine blue findings and adjusted for confounders, as both are associated with MT in the literature but without a North American‐specific cohort analysis. Our primary model included both variables of interest. A secondary model included only anatomic site since toluidine blue is not in widespread use.

**Results:**

Five hundred and thirty‐four participants with 605 lesions met final inclusion criteria, with 339 mild and 266 moderate OED at baseline. In the primary model, lesions at a high‐risk anatomic site or with positive toluidine blue staining were associated with a 2.6 and 2.4‐fold increased risk of progression, respectively. In the second model that did not incorporate toluidine blue, high‐risk anatomic site remained a highly associated risk factor (2.7‐fold increased risk of progression).

**Conclusion:**

Lesion anatomic site is associated with higher risk of MT for the general practitioner, while a specialist with access to toluidine blue results can assume additional risk associated with positive staining. These models may inform decisions for surveillance and intervention for OED.

## 1 Introduction

In 2022, the Global Cancer Observatory (GLOBOCAN) reported that almost 390 000 cases of lip and oral cavity cancer were diagnosed worldwide, with 188 000 associated deaths [1], and greater than 90% of these cancers are oral squamous cell carcinomas (OSCC). The histopathological presence of oral epithelial dysplasia (OED) remains the current gold standard for assessing the risk of progression to carcinoma in patients presenting with oral potentially malignant disorders (OPMD) such as leukoplakia or erythroplakia, and the higher the degree of dysplasia, the greater the risk of progression [2]. OED is graded as mild, moderate, or severe (including carcinoma in situ (CIS)). Mild and moderate dysplasia (low‐grade OED) involves changes in the lower third and middle third of the epithelial thickness, respectively, while severe dysplasia/CIS (high‐grade OED) involves changes in more than two‐thirds [3]. The rate of malignant transformation (MT) of OED varies across studies, but a meta‐analysis reported an overall rate of MT for OED of 12.1%, with a mean time to MT of 4.3 years [4]. The risk of MT in patients with lesions harboring severe dysplasia/*CIS* is high, and hence these lesions are sent for curative surgical treatment prior to MT. Low‐grade OED is more prevalent and has a comparatively lower MT rate than high‐grade OED, and it remains a widespread challenge for clinicians to differentiate which patients with low‐grade OED merit closer monitoring or intervention. More definitive risk assessment would improve clinical decision‐making for these lesions.

Studies have investigated the clinical features associated with a risk of progression in patients with leukoplakia and erythroplakia with (or without) OED, although most are retrospective studies. These include lesion color, anatomic site, size, and topographical appearance [5, 6, 7, 8]. The floor of the mouth and the ventral and lateral borders of the tongue are thought to be high‐risk sites for MT, theorized to be related to carcinogens from tobacco and alcohol pooling in saliva [2, 9]. Toluidine blue (TB) is a vital stain‐based diagnostic adjunctive technique, and positive TB staining has been strongly associated with the presence of high‐grade OED and OSCC [10], and also thought to portend increased risk for MT in low‐grade OED, though with limitations of small sample size and univariable analysis and in particular a paucity of representation of North American populations [11].

To date, there is a paucity of models using real‐world data from large prospective longitudinal cohorts supporting reliable indicators of risk of progression in OED [12]. Predictive tissue biomarkers for progression risk, such as loss of heterozygosity (LOH) and aneuploidy, have been validated in such prospective studies, but they are not readily available for commercial use by most clinicians [13, 14, 15]. To date, there are no models that allow risk to be estimated based on factors routinely collected by clinicians in primary and/or secondary care settings. In this paper, we present clinically applicable models to aid in determining the risk of progression in patients with lesions harboring mild or moderate (low‐grade) OED using demographic, risk habit, and clinical features.

## 2 Materials and Methods

### 2.1 Study Population

All patients in this study were enrolled in the ongoing Oral Cancer Prediction Longitudinal (OCPL) study in Vancouver, British Columbia, Canada, between January 1, 1997 and December 31, 2021. Patients diagnosed with OED are referred from community practices in the province of British Columbia to the BC Oral Cancer Prevention Program's oral dysplasia clinics (NextGen Dysplasia Clinics) in the Greater Vancouver area. All data collected (demographic, medical, clinical, histological, and biomarkers) were entered into a database. Inclusion requirements for the current study included a lesion with biopsy‐proven mild or moderate OED and no previous high‐grade OED or OSCC. Ethical approval was obtained from the University of British Columbia/BC Cancer Agency Research Ethics Board, and participants were accrued to the study using written informed consent.

### 2.2 Data Collection

The OCPL study collects demographic information and risk habit information on standardized assessment forms. Tobacco and alcohol use are updated annually and include the amount and duration of habit use, along with start and quit dates. A smoker was defined as having smoked more than 100 cigarettes or the equivalent in their lifetime [16]. Alcohol consumption was recorded as the average weekly number of drinks, where one alcoholic drink equaled a 12 oz. beer, 5 oz. of wine, or 1 oz. of spirits [17].

Clinical features recorded in this study (anatomic site, size, color, texture, appearance, and TB status) were collected prospectively at study entry and at 6‐month intervals. Appearance was recorded as either homogeneous (uniformly white color and smooth textured throughout the lesion) or non‐homogeneous (not uniformly white and/or smooth). Clinical data were collected for each lesion if multiple dysplastic lesions were present. Anatomic site was categorized as high‐risk (floor of mouth and ventral and lateral borders of the tongue) and low‐risk (all other oral sites). “No lesion” was recorded if a former biopsy site had no visible lesion or only a scar.

### 2.3 Missing Data

Several of our predictor variables had missing values, which we assume are missing at random (MAR) (conditional on the covariates in our model). In order to retain all observations for our models, we imputed these missing values using the imputation algorithm from the “randomForestSRC” package [18]. This implements a random survival forest‐based imputation algorithm developed by Ishwaran et al. [19]. (More details are given in the Supporting Information) Using the resulting imputed data, we proceeded to model fitting.

Clinical images of lesions were taken at each study visit. Biopsies were taken every 2 years or when there were concerning clinical changes, and histopathological results were recorded. All lesions with low‐grade OED were included for analysis, and the study's primary disease endpoint was when the lesion (or the first lesion in patients with multiple lesions) progressed to either severe dysplasia/CIS or OSCC (or verrucous carcinoma (VC)). Severe dysplasia was included in the progressors' outcome group based on evidence reported by our group and others that severe dysplasia progresses to carcinoma at a high rate of approximately 32% of patients in 3 years and 59% in 5 years [20]. All patients that progressed were sent for surgical excision or resection. Patients whose lesion(s) did not progress were denoted as non‐progressors. The time of follow‐up was defined as from the date of initial biopsy to the date of progression (progressors) or last follow‐up visit (non‐progressors).

### 2.4 Statistical Models

#### 2.4.1 Inference

To model our time‐to‐event data, we fit Cox proportional hazards models using the ‘survival’ package in R [18]. The response variable was the time to progression to high‐grade OED or carcinoma (OSCC/VC) from the time of the first biopsy. The primary objective was to estimate the effects of anatomic site and TB on the time to progression. Therefore, our primary model estimated the effects of both anatomic site and TB simultaneously in a Cox model (along with all confounders). We also fit a secondary model that estimated only anatomic site (along with all confounders) because TB is typically used in expert settings, and a model with the other features may be useful for the primary setting. Exploratory Kaplan–Meier analyses suggested that both TB and anatomic site were associated with a higher risk of progression, justifying further investigation in more complex models. In this context, “adjusted” means that our analysis accounts for the influence of other variables that could affect the relationship between anatomic site, TB, and disease progression, ensuring that the results specifically reflect the independent effects of the primary variables of interest. Therefore, we included several suspected confounding variables in addition to these two primary predictor variables. This allowed us to estimate an adjusted hazard ratio (HR) for each of anatomic site and TB, accompanied by 95% confidence intervals (CIs), using our Cox models. Specifically, the primary model included TB status at initial visit, anatomic site, the presence of multiple sites, sex, age at first diagnosis of dysplasia, smoking frequency and duration, alcohol use, race, lesion area, and color and texture (appearance). The second model included all the parameters of model one except for TB status.

To account for the existence of multiple lesions within individuals, robust sandwich type standard errors quantify the uncertainty in our parameter estimates.

We evaluated the proportional hazards assumption, specifically by testing the null hypothesis that there is no correlation between the Schoenfeld residuals and time. A zero slope in the fitted regression line is consistent with no violations in the proportional hazards assumption.

We then constructed estimated survival curves using our primary fitted Cox model for a hypothetical patient with baseline covariates and a hypothetical at‐risk subject. To do so, we used a fitted Cox model to estimate survival probabilities at various time points.

## 3 Results

### 3.1 Demographic, Risk Habit and Clinical Features

There were 605 low‐grade OED lesions originating from 534 unique patients that met study criteria: 472 of the patients had one lesion under observation in the study; 55 patients had two lesions; 6 patients had three lesions; and 1 patient had five lesions. Among the OED lesions, 339 were graded as mild dysplasia and 266 were moderate dysplasia. Forty patients, accounting for 49 lesions, died in follow‐up. Descriptive statistics for demographic and risk habits and outcomes are shown in Table 1, while clinical variables and outcomes are in Table 2. The mean time to progression was 36.2 months (SD: 29.1 months, median 32 months), while non‐progressing lesions had follow‐up for a mean of 61.2 months (SD: 44.6 months, median 54 months).

**TABLE 1:** Distribution of sociodemographic and smoking and alcohol status and progression status.

	Lesions		
	All	Non‐progressors	Progressors
	*N* (%)	*N* (%)	*N* (%)
	605	555 (92)	50 (8)
Age at diagnosis years, mean (SD)	59.1 (11.7)	59.2 (11.7)	57.4 (11.5)
Age category (years)			
< 50	133 (22)	117 (21)	16 (32)
50–59	178 (29)	166 (30)	12 (24)
60–69	171 (29)	159 (29)	12 (24)
≥ 70	123 (20)	113 (20)	10 (20)
Sex			
Female	267 (44)	242 (44)	25 (50)
Male	338 (56)	313 (56)	25 (50)
Race			
White	502 (83)	464 (84)	38 (76)
Asian	86 (14)	76 (14)	10 (20)
First nations	3 (< 1)	3 (< 1)	0
Black	4 (< 1)	4 (< 1)	0
Other	10 (2)	8 (1)	2 (4)

Lifetime average frequency (cigs/day)^a^	Lifetime duration (years)	Lesions		
		All	Non‐progressors	Progressors
		*N* (%)	*N* (%)	*N* (%)
Smoking status (*N* = 592)				
Never^b^	Never	206 (34)	185 (33)	21 (42)
≤ 20	≤ 20	57 (9)	54 (10)	3 (6)
≤ 20	> 20	85 (14)	77 (14)	8 (16)
> 20	≤ 20	17 (3)	13 (2)	4 (8)
> 20	> 20	227 (38)	214 (39)	13 (26)
Missing values		13 (2)	12 (2)	1 (2)
Alcohol status (drinks/week)^c^ (*N* = 571)				
0–6		332 (55)	303 (55)	29 (58)
7–20		158 (26)	144 (26)	14 (28)
≥ 21		81 (13)	75 (14)	6 (12)
Missing values		34 (6)	33 (6)	1 (2)

^a^ Cigs/day weighted over one's lifetime.

^b^ Never smoker defined as less than 100 cigarettes in lifetime.

^c^ Years drank weighted over one's lifetime.

**TABLE 2:** Distribution of clinical features of diagnosed with OED and progression status.

	All	Non‐progressors	Progressors
	*N* (%)	*N* (%)	*N* (%)
	(*N* = 605)	(*N* = 555, 92%)	(*N* = 50, 8%)
Color			
White only	271 (45)	252 (45)	19 (38)
Other	235 (39)	205 (37)	30 (60)
No lesion^a^	76 (13)	76 (14)	0
Missing values	23 (4)	22 (4)	1(2)
Appearance			
Homogeneous	185 (31)	173 (31)	12 (24)
Non‐homogeneous	321 (53)	284 (51)	37 (74)
No lesion^a^	77 (13)	77 (14)	0
Missing values	22 (4)	21 (4)	1 (2)
Size			
≤ 200 mm^2^	334 (55)	316 (57)	18 (36)
> 200 mm^2^	244 (40)	213 (38)	31 (62)
Missing values	27 (4)	26 (5)	1 (2)
Anatomic site^b^			
Low risk	282 (47)	268 (48)	14 (28)
High risk	323 (53)	287 (52)	36 (72)
Number of sites^c^			
1	362 (60)	330 (59)	32 (64)
≥ 2	243 (40)	225 (41)	18 (36)
Toluidine blue			
Negative	509 (84)	476 (86)	33 (66)
Positive	63 (10)	50 (9)	13 (26)
Equivocal	12 (2)	10 (2)	2 (4)
Not done	10 (2)	8 (1)	2 (4)
Missing values	11 (2)	11 (2)	0
Dysplasia grade			
Mild	339 (56)	324 (58)	15 (30)
Moderate	266 (44)	231 (42)	35 (70)

^a^ Scar or no lesion was visible.

^b^ High‐risk site—lateral, ventral tongue, and floor of mouth.

^c^ A lesion was considered a separate site if it was at least three centimeters away from another lesion.

### 3.2 Model Development

In the primary model, anatomic site and positive TB staining were both estimated to be highly associated with the risk of progression (Table 3). The estimated hazard ratios and accompanying 95% confidence intervals (CIs) for a high‐risk anatomic site and positive TB staining were 2.6 (1.3–5.1) and 2.4 (1.1–5.1), respectively. Conditional on the effects of the other predictors (i.e., potential confounders), a greater than twofold increase in the risk of progression from mild or moderate dysplasia to severe dysplasia/CIS or OSCC/VCC was observed for the anatomic site of a lesion and a positive TB result. In addition, appearance, in terms of homogeneous color and texture of lesions, was estimated to be highly associated with progression (Table S3).

**TABLE 3:** Association between TB status, anatomic site, and risk of progression.

Parameter	Model 1^a^		Model 2^b^	
	HR (95% CI)	*p*	HR (95% CI)	*p*
TB negative^c^	1.0			
TB positive^c^	2.4 (1.1–5.1)	**0.020**	—	—
TB equivocal	2.2 (0.5–10.1)	0.298	—	—
TB not done	3.7 (0.7–19.4)	0.126	—	—
Low‐risk anatomic site	1.0		1.0	
High‐risk anatomic site*	2.6 (1.3–5.1)	**0.008**	2.7 (1.3–5.4)	**0.006**

*Note*: Bold values indicate statistically significant (*p* < 0.05).

Abbreviations: 95% CI, 95% confidence interval; HR, hazard ratio.

^a^ Includes key risk factors and potential confounders: presence of multiple sites, sex, age at first diagnosis of dysplasia, smoking frequency and duration, alcohol use, race, lesion area, and color and texture (appearance).

^b^ Model 2 excludes TB.

^c^ TB positive, lesion picked up stain; TB equivocal, lesion staining is inconclusive; TB negative (reference group), the lesion did not pick up stain.

In the secondary model that omitted TB, the estimated hazard ratio and 95% CI for a high‐risk anatomic site was 2.7 (1.3–5.4), nearly identical to the estimate in model 1, and consistent with the hypothesis that TB provides additional explanatory information for the probability of progression.

**FIGURE 1:**
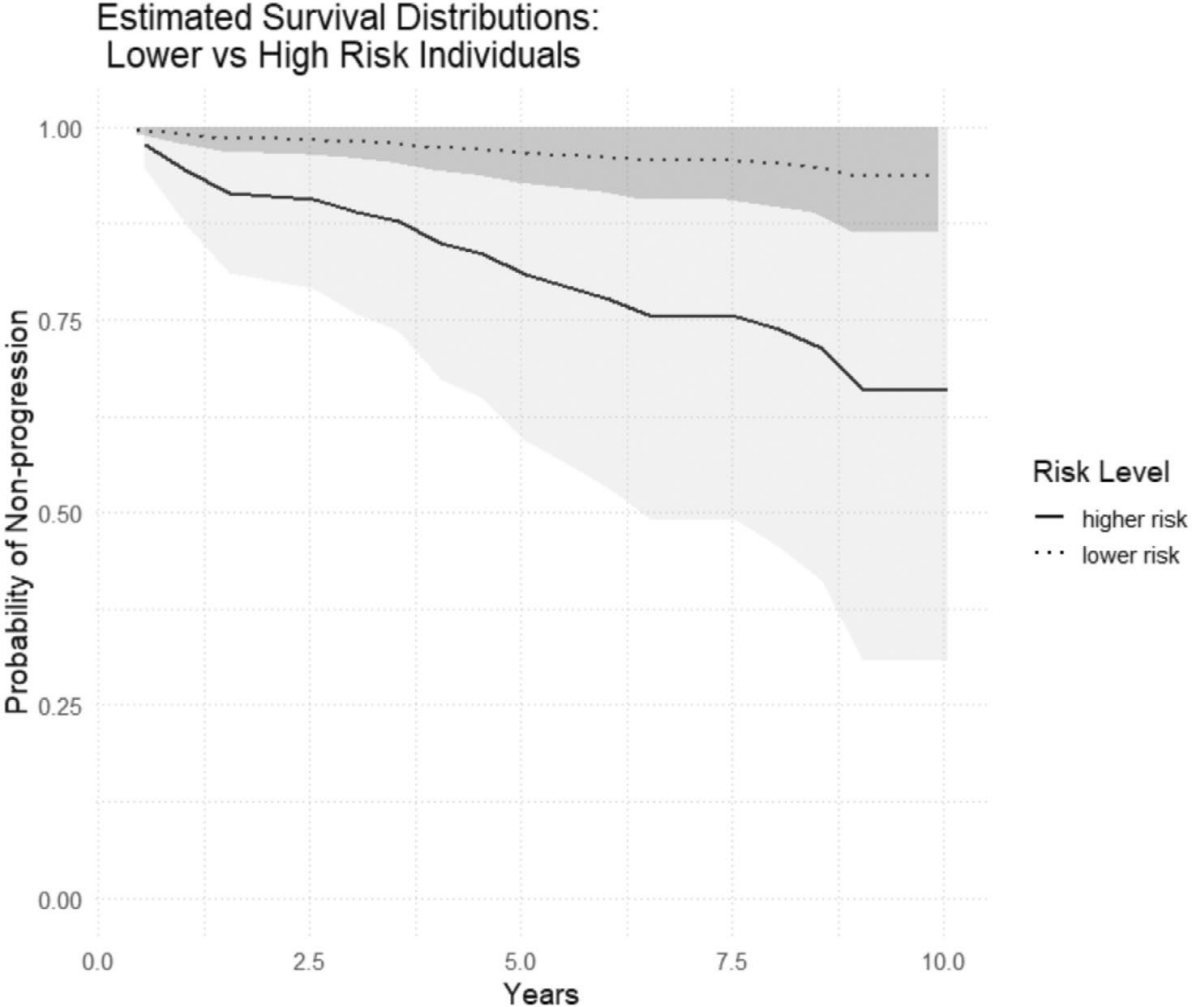
Estimated survival curves and pointwise 95% confidence intervals (shading) comparing a higher risk vs. lower risk anatomic site individuals. The higher risk curve corresponds to a lesion located at an anatomic site more prone to progression and with positive TB staining. The lower risk curve corresponds to a lesion with negative TB staining and located at anatomic site less prone to progression. All other covariates were set at their baseline values: Single site (no multiple sites), female sex, age < 50 years, smoking frequency ≤ 20 cigs/day and duration ≤ 20 years, alcohol consumption of 0–6 units per week, white (race), lesion area < 200 mm^2^, and homogeneous appearance.

Estimated survival curves for low and relatively high‐risk hypothetical subjects, respectively, from 0.5 to 10 years after the initial clinical visit showed clear separation of the estimated curves (Figure 1).

## 4 Discussion

The identification of patients with lesions harboring low‐risk OEDs at increased risk for progression by clinicians is of paramount importance in the prevention of OSCC. Theoretically, once a dysplastic lesion is found to be at an increased risk for progression, clinical interventions can be recommended to remove or mitigate risk. The use of clinical prediction models to aid in the assessment of the risk of progression has not been adopted in standard care, though it has the potential to guide decision‐making for clinicians who monitor patients with lesions harboring low‐grade dysplasia. All patients with abnormal findings on a visual and tactile exam that are deemed clinically suspicious for OSCC or OPMDs are recommended for biopsy. If the definitive histopathologic diagnosis is low‐grade OED, the decision to treat or monitor can be challenging, largely due to the lack of agreement on treatment for OED [21].

OED remains the current gold standard predictor for progression and typically guides clinicians in the management of this patient population. Yet, as a single predictor, histopathology is imperfect due to limitations of sampling and pathologist agreement. Importantly, the definitive diagnosis of dysplasia rendered by an oral pathologist, particularly in the context of mild and moderate dysplasia, can be subjective and is unpredictable as an indicator of risk [21]. OED is a disorder diagnosed by the identification of 28 cytological and architectural changes in the oral epithelium [3, 21]. In a meta‐analysis of the MT of OPMD, Guan et al. [22] found that the risk of MT versus non‐dysplastic leukoplakia increased progressively with the severity of OED: mild OED showed a 1.87‐fold increased risk, moderate OED a 2.92‐fold increased risk, and severe OED a 7.13‐fold increased risk. Conversely, Arduino et al. [23], in their study of 207 patients with OED, found that the grade of dysplasia was not associated with MT. Inclusion of severe dysplasia in the sample can also influence MT rates. In retrospective analyses of OED that included low‐ and high‐grade OED, Kierce et al. [24], Jaber and Elameen [25] and Gomez‐Armayones et al. [26] and had MT rates of 16.7%, 5.5%, and 42%, respectively. In a systematic review, Mehanna et al. [4] reported a MT rate of 10.3% for low‐grade dysplasia. In our study, where severe dysplasia is an endpoint, the progression rate was 8% with a larger proportion of moderate OED (13%) having progressed than mild OED (4%).

Various clinical characteristics have been found to be associated with an increased risk of MT in OPMDs, with and without OED. These clinical characteristics include OPMDs in females and non‐smokers, the anatomic site of the lesion (oral tongue or floor of mouth), larger size (> 2 cm^2^), a non‐homogeneous appearance, and the presence of OED [6, 9]. However, only a few of these studies look at a multivariate relationship between such factors and MT. In a longitudinal study of patients with OED by Ho et al. [6], 25% of lesions harboring OED underwent MT, and a multivariate analysis found that non‐smokers and a non‐homogeneous clinical appearance were at the greatest risk of MT. The authors attributed the higher MT rate to both the higher prevalence of patients with severely dysplastic lesions and a long follow‐up time. Similarly, Arduino et al. [23] found that non‐homogeneous appearance was the only clinical factor associated with MT. Our multivariable modeling determined that high‐risk anatomic site and TB positive status were associated with progression.

Toluidine blue has a long history (> 50 years) as a diagnostic adjunctive technique for detecting OSCC, but its use in OED has been controversial [27]. Interpretation can be subjective, and the possibility exists for false positive and false negative staining. Interpretation requires expertise and experience. As such, an expert panel convened by the American Dental Association has recommended against the use of vital staining by general dentists. TB is an acidophilic vital stain that works by binding the phosphate groups of nucleic acids, which are found in higher concentrations in cancer and progressing neoplastic tissue [28]. In addition, it penetrates between the cells of neoplastic tissue due to defective intercellular barriers [29]. In an expert setting, however, TB has been demonstrated to have utility to facilitate biopsy site selection and to identify satellite lesions [30]. Zhang et al. [11] found TB positivity to be associated with a four‐fold increased risk of MT for low‐grade dysplasia. The authors also reported TB positivity was associated with a non‐homogeneous appearance, large size, and predictive molecular markers for MT (i.e., loss of heterozygosity (LOH)). In this study, we demonstrated in multivariable analysis that TB positivity is associated with progression.

This study was limited by the relatively small number of lesions with clinical progression and the possibility that results may not apply to patients with a prior history of high‐grade OED as these patients were excluded. Still, the data for this study were collected prospectively in consecutively referred patients using standardized forms, all patients had biopsy‐proven dysplasia, and dysplasia was followed regularly by clinicians trained by the same team, and all biopsies for this study were reviewed by the same oral pathology team.

In conclusion, we examined a large clinical dataset, using a robust multivariable analysis, the association of anatomic site and TB with malignant transformation for risk assessment of patients with low‐grade OED to assist with decisions on close surveillance and early intervention. These statistical models were fit using demographic, risk habit, and clinical features from a long‐term prospective study of British Columbia patients with low‐grade OED lesions. This led to two models with both anatomical site and TB that can be easily integrated into clinical care, the first model to assist clinicians with TB expertise, while the second model excluded TB to assist clinicians in any setting that do not utilize TB. Given the lack of commercially available real‐time chairside adjunctive techniques built on the validation of novel “‐omics” biomarkers, we feel that these simple models can improve risk stratification and facilitate important treatment decisions. This second model found that high‐risk anatomical site maintained a strong association with progression. In a primary care setting where most clinicians do not routinely use toluidine blue, patients with mild OED involving high‐risk sites would be referred to secondary “expert setting” where the use of toluidine blue is more commonly employed. It is important to note that OSCC can occur at any oral site and OED should be followed closely regardless of site and TB status. However, clinicians encountering patients diagnosed with lesions with low‐grade dysplasia at high‐risk anatomic sites, and with positive TB staining also, might consider a more aggressive approach to intervention, such as surgical excision, chemoprevention, or more frequent surveillance to prevent or mitigate the risk for progression.

## Author Contributions

**Denise M. Laronde:** data curation, resources, project administration, supervision, investigation, visualization, writing – original draft. **Matt Berkowitz:** methodology, formal analysis, visualization, writing – original draft. **A. Ross Kerr:** visualization, methodology, writing – review and editing. **Erinn M. Hade:** methodology, formal analysis, writing – review and editing. **Mutita Siriruchatanon:** writing – review and editing. **Miriam P. Rosin:** data curation, writing – review and editing. **Stella K. Kang:** funding acquisition, conceptualization, visualization, project administration, supervision, methodology, writing – review and editing.

## Ethics Statement

This study was reviewed and approved by the University of British Columbia Research Ethics Board and New York University Institutional Review Board.

## Conflicts of Interest

The authors declare no conflicts of interest.

## Supporting information


**Data S1:** Supporting Information.

## Data Availability

The data that support the findings of this study are available on request from the corresponding author. The data are not publicly available due to privacy or ethical restrictions.

## References

[1] F. Bray , M. Laversanne , H. Sung , et al., “Global Cancer Statistics 2022: GLOBOCAN Estimates of Incidence and Mortality Worldwide for 36 Cancers in 185 Countries,” CA: A Cancer Journal for Clinicians 74, no. 3 (2024): 229–263. 38572751 10.3322/caac.21834

[2] P. M. Speight , S. A. Khurram , and O. Kujan , “Oral Potentially Malignant Disorders: Risk of Progression to Malignancy,” Oral Surgery, Oral Medicine, Oral Pathology, Oral Radiology 125, no. 6 (2018): 612–627. 29396319 10.1016/j.oooo.2017.12.011

[3] S. Muller and W. M. Tilakaratne , “Update From the 5th Edition of the World Health Organization Classification of Head and Neck Tumors: Tumours of the Oral Cavity and Mobile Tongue,” Head and Neck Pathology 16, no. 1 (2022): 54–62. 35312982 10.1007/s12105-021-01402-9 PMC9018914

[4] H. M. Mehanna , T. Rattay , J. Smith , and C. C. McConkey , “Treatment and Follow‐Up of Oral Dysplasia ‐ A Systematic Review and Meta‐Analysis,” Head & Neck 31, no. 12 (2009): 1600–1609. 19455705 10.1002/hed.21131

[5] S. Silverman , M. Gorsky , and F. Lozada , “Oral Leukoplakia and Malignant Transformation,” Cancer 53 (1984): 563–568. 6537892 10.1002/1097-0142(19840201)53:3<563::aid-cncr2820530332>3.0.co;2-f

[6] M. W. Ho , J. M. Risk , J. A. Woolgar , et al., “The Clinical Determinants of Malignant Transformation in Oral Epithelial Dysplasia,” Oral Oncology 48, no. 10 (2012): 969–976. 22579265 10.1016/j.oraloncology.2012.04.002

[7] F. Dost , K. A. Le Cao , P. J. Ford , et al., “A Retrospective Analysis of Clinical Features of Oral Malignant and Potentially Malignant Disorders With and Without Oral Epithelial Dysplasia,” Oral Surgery, Oral Medicine, Oral Pathology, Oral Radiology 116, no. 6 (2013): 725–733. 24144993 10.1016/j.oooo.2013.08.005

[8] L. D. Rock , M. P. Rosin , L. Zhang , B. Chan , B. Shariati , and D. M. Laronde , “Characterization of Epithelial Oral Dysplasia in Non‐Smokers: First Steps Towards Precision Medicine,” Oral Oncology 78 (2018): 119–125. 29496039 10.1016/j.oraloncology.2018.01.028 PMC5836799

[9] S. Warnakulasuriya and A. Ariyawardana , “Malignant Transformation of Oral Leukoplakia: A Systematic Review of Observational Studies,” Journal of Oral Pathology & Medicine 45, no. 3 (2016): 155–166. 26189354 10.1111/jop.12339

[10] D. H. Kim , E. A. Song , S. W. Kim , and S. H. Hwang , “Efficacy of Toluidine Blue in the Diagnosis and Screening of Oral Cancer and Pre‐Cancer: A Systematic Review and Meta‐Analysis,” Clinical Otolaryngology 46, no. 1 (2021): 23–30. 32741142 10.1111/coa.13613

[11] L. Zhang , M. Williams , C. F. Poh , et al., “Toluidine Blue Staining Identifies High‐Risk Primary Oral Premalignant Lesions With Poor Outcome,” Cancer Research 65, no. 17 (2005): 8017–8021. 16140975 10.1158/0008-5472.CAN-04-3153

[12] I. van der Waal , “Potentially Malignant Disorders of the Oral and Oropharyngeal Mucosa; Present Concepts of Management,” Oral Oncology 46, no. 6 (2010): 423–425. 20308005 10.1016/j.oraloncology.2010.02.016

[13] L. Zhang , C. F. Poh , M. Williams , et al., “Loss of Heterozygosity (LOH) Profiles: Validated Risk Predictors for Progression to Oral Cancer,” Cancer Prevention Research 5, no. 9 (2012): 1081–1089. 22911111 10.1158/1940-6207.CAPR-12-0173 PMC3793638

[14] M. Datta , D. M. Laronde , M. P. Rosin , L. Zhang , B. Chan , and M. Guillaud , “Predicting Progression of Low‐Grade Oral Dysplasia Using Brushing‐Based DNA Ploidy and Chromatin Organization Analysis,” Cancer Prevention Research 14, no. 12 (2021): 1111–1118. 34376461 10.1158/1940-6207.CAPR-21-0134 PMC8639617

[15] E. W. Odell , “Aneuploidy and Loss of Heterozygosity as Risk Markers for Malignant Transformation in Oral Mucosa,” Oral Diseases 27, no. 8 (2021): 1993–2007. 33577101 10.1111/odi.13797

[16] S. J. Bondy , J. C. Victor , and L. M. Diemert , “Origin and Use of the 100 Cigarette Criterion in Tobacco Surveys,” Tobacco Control 18, no. 4 (2009): 317–323. 19491091 10.1136/tc.2008.027276

[17] E. A. Field , T. Morrison , A. E. Darling , T. A. Parr , and J. M. Zakrzewska , “Oral Mucosal Screening as an Integral‐Part of Routine Dental‐Care,” British Dental Journal 179, no. 7 (1995): 262–266. 7577180 10.1038/sj.bdj.4808894

[18] R Core Team , R: A Language and Environment for Statistical Computing (R Foundation for Statistical Computing, 2023).

[19] H. Ishwaran , U. B. Kogalur , E. H. Blackstone , and M. S. Lauer , “Random Survival Forests,” Annals of Applied Statistics 2, no. 3 (2008): 841–860.

[20] L. Zhang , T. Lubpairee , D. M. Laronde , and M. P. Rosin , “Should Severe Epithelial Dysplasia Be Treated?,” Oral Oncology 60 (2016): 125–129. 27531883 10.1016/j.oraloncology.2016.07.007 PMC4991622

[21] P. Hankinson , H. Mahmood , H. Walsh , P. M. Speight , and S. A. Khurram , “Demystifying Oral Epithelial Dysplasia: A Histological Guide,” Pathology 56, no. 1 (2024): 11–23. 38030478 10.1016/j.pathol.2023.10.002

[22] J. Y. Guan , Y. H. Luo , Y. Y. Lin , et al., “Malignant Transformation Rate of Oral Leukoplakia in the Past 20 Years: A Systematic Review and Meta‐Analysis,” Journal of Oral Pathology & Medicine 52, no. 8 (2023): 691–700. 37224426 10.1111/jop.13440

[23] P. G. Arduino , A. Surace , M. Carbone , et al., “Outcome of Oral Dysplasia: A Retrospective Hospital‐Based Study of 207 Patients With a Long Follow‐Up,” Journal of Oral Pathology & Medicine 38, no. 6 (2009): 540–544. 19453839 10.1111/j.1600-0714.2009.00782.x

[24] J. Kierce , Y. Shi , H. Klieb , N. Blanas , W. Xu , and M. Magalhaes , “Identification of Specific Clinical Risk Factors Associated With the Malignant Transformation of Oral Epithelial Dysplasia,” Head & Neck 43, no. 11 (2021): 3552–3561. 34472151 10.1002/hed.26851

[25] M. A. Jaber and E. M. Elameen , “Long‐Term Follow‐Up of Oral Epithelial Dysplasia: A Hospital Based Cross‐Sectional Study,” Journal of Dental Science 16, no. 1 (2021): 304–310. 10.1016/j.jds.2020.04.003 PMC7770253 33384813

[26] S. Gomez‐Armayones , E. Chimenos‐Kustner , C. Arranz , et al., “Risk Factors for Oral Epithelial Dysplasias to Become Malignant: Clinical Implications,” International Journal of Oral and Maxillofacial Surgery 51, no. 4 (2022): 473–480. 34503889 10.1016/j.ijom.2021.08.012

[27] K. A. S. Warnakulasuriya and N. W. Johnson , “Sensitivity and Specificity of OroScan Toluidine Blue Mouthrinse in the Detection of Oral Cancer and Precancer,” Journal of Oral Pathology & Medicine 25 (1996): 97–103. 9148038 10.1111/j.1600-0714.1996.tb00201.x

[28] A. J. Dunipace , R. Beaven , T. Noblitt , L. Yiming , S. Zunt , and G. Stookey , “Mutagenic Potential of Toluidine Blue Evaluated in the Ames Test,” Mutation Research 279, no. 4 (1992): 255–259. 1377780 10.1016/0165-1218(92)90241-q

[29] P. Herlin , J. Marnay , J. H. Jacob , et al., “A Study of the Mechanism of the Toluidine Blue Dye Test,” Endoscopy 15, no. 1 (1983): 4–7. 6185331 10.1055/s-2007-1018595

[30] D. Rosenberg and S. Cretin , “Use of Meta‐Analysis to Evaluate Tolonium Chloride in Oral Cancer Screening,” Oral Surgery, Oral Medicine, and Oral Pathology 67, no. 5 (1989): 621–627. 2654801 10.1016/0030-4220(89)90286-7

