# Supplementary material for: Clinical Features Associated With Malignant Transformation of Low‐Grade Dysplasia
Source: J Oral Pathol Med. 2025 Oct 6;55(1):99–106. doi: 10.1111/jop.70070 (PMC12774577; doi:10.1111/jop.70070)
Supplement: Supplementary file 1 — Data S1: Supporting Information. [file JOP-55-99-s001.docx]

**Supplemental data**

| **Table 3 expanded.** Hazard ratios and p-values for Cox models 1 and 2 | | | | | | |
| --- | --- | --- | --- | --- | --- | --- |
|  | **Model 1^†^** | |  |  | **Model 2^‡^** | |
|  |  |  |  |  |  |  |
| **Parameter** | **HR(95% CI)** | ***P*** |  |  | **HR(95% CI)** | ***P*** |
| TB Positive^§^ | 2.4 (1.1–5.1) | **0.020** |  |  | - | - |
| TB Equivocal | 2.2 (0.5–10.1) | 0.298 |  |  | - | - |
| TB Not Done | 3.7 (0.7–19.4) | 0.126 |  |  | - | - |
| High risk site | 2.6 (1.3–5.1) | **0.008** |  |  | 2.7 (1.3–5.4) | **0.006** |
| Appearance: Non-Homogeneous | 2.4 (1.1–4.9) | **0.023** |  |  | 2.8 (1.4–5.9) | **0.005** |
| Area: ≥200mm^2^ | 1.5 (0.8–2.8) | 0.195 |  |  | 1.7 (0.9–3.1) | 0.083 |
| Multiple Sites: Yes | 0.6 (0.3–1.2) | 0.162 |  |  | 0.7 (0.3–1.3) | 0.221 |
| Sex: Female | 1.0 (0.6–1.9) | 0.944 |  |  | 1.0 (0.6–1.9) | 0.909 |
| Age (years): ≥50, <60 | 0.5 (0.2–1.2) | 0.107 |  |  | 0.5 (0.2–1.2) | 0.114 |
| Age (years): ≥60, <70 | 0.6 (0.3–1.3) | 0.179 |  |  | 0.6 (0.3–1.3) | 0.227 |
| Age (years): ≥70 | 0.6 (0.2–1.5) | 0.255 |  |  | 0.6 (0.3–1.5) | 0.300 |
| SmkFreqDur: Never/Never | 0.6 (0.2–2.1) | 0.477 |  |  | 0.6 (0.2–2.2) | 0.463 |
| SmkFreqDur: ≤20/day+≤20 years^¶^ | 1.2 (0.5–3.1) | 0.732 |  |  | 1.1 (0.4–2.8) | 0.848 |
| SmkFreqDur: ≤20/day+>20 years | 3.3 (0.8–14.1) | 0.103 |  |  | 3.7 (1.1–12.7) | **0.035** |
| SmkFreqDur: >20/day+>20 years | 0.9 (0.4–2.0) | 0.850 |  |  | 0.9 (0.4–1.9) | 0.774 |
| Alcohol: ≤6 drinks/week^#^ | 1.0 (0.5–1.9) | 0.903 |  |  | 0.9 (0.5–1.9) | 0.858 |
| Alcohol: >6 - ≤20 drinks/week | 0.8 (0.3–2.3) | 0.711 |  |  | 0.8 (0.3–2.2) | 0.698 |
| Ethnicity: NonWhite | 1.6 (0.8–3.2) | 0.212 |  |  | 1.6 (0.8–3.2) | 0.224 |
| ^†^ includes all predictors.  ^‡^ includes all predictors except TB.  ^§^ TB positive = lesion picked up stain; TB equivocal = lesion staining is inconclusive; TB negative = the lesion did not pick up stain.  ^¶^ SmkFreqDur = smoking frequency and duration. Cigs/day weighted over one's lifetime.  ^#^ Years drank weighted over one's lifetime. | | | | | | |

The above table shows the estimated effects of all covariates in our primary model. The top section shows the estimates of our variables of interest (TB and Anatomic site), while the bottom section shows the estimates of our confounders. Among the confounders, Appearance and SmkFreqDur: ≤20/day + >20 years were significant in explaining time to progression.

**Imputation of Missing Data:**

Here we provide more details on how we implemented the imputation algorithm via random survival forests (RSFs). The RSF-based algorithm uses an iterative process to impute missing values. The initial step randomly fills in missing values using the empirical distribution function derived from the non-missing data in each node. The node is then split using this imputed data. After splitting, the imputed values in the daughter nodes are reset to missing, and the process is repeated. This cycle continues until the survival tree is fully grown. Multiple forests are grown iteratively, each time using the imputed data from the previous forest, until the changes in imputed values between iterations fall below a certain threshold, ensuring accurate and robust results.

Given that the proportion of missing data was very small, we elected not to use a multiple imputation method. Though we did not use multiple imputation, we ran several sensitivity checks to ensure the reliability of our model estimates. We compared the estimates from Cox models using our fully imputed dataset with a complete cases dataset (in which any observations with missing data were deleted). The estimated effects of the covariates and the confidence intervals (for these effects) were very similar using both the imputed and complete cases datasets, indicating that our imputation algorithm likely did not substantively affect the results.

**Calibration of Prediction Model**

For our prediction model, we reported on the C-index (our *discrimination* metric) to assess its performance in one respect. We also considered the integration calibration index (ICI), a *calibration* metric to assess performance in another respect. The ICI measures the difference between predicted probabilities and observed probabilities via a smoothed calibration curve—0 denoting perfect fit and 1 denoting the worst possible score. We fit restricted cubic splines using 3 knots to compute the mean 3-year ICI using 1000 random splits of the data—80% of which was used for the training set, and the remaining 20% for the test set, similarly to how we computed the C-index. Though the mean ICI (over the 1000 splits) was reasonable, the lack of event time data led to the ICI figures varying widely, with many ICI values (on a single partition of the data) lying close to 1.0. This result offers additional support against using our prediction models to guide clinical decision-making on individual patients.

**Implementation:**

All programming was done in R. We imputed missing values using the ‘impute.rfsrc()’ function from the ‘randomForestSRC’ package. We used the the ‘coxph()’ function from the ‘survival’ package to fit the Cox models. We tested the proportional hazards assumption using the ‘cox.zph()’ function. To construct the estimated survival curves, we used the ‘predict’ function on the fitted Cox model to estimate survival probabilities at various time points. We used the ‘rms’, ‘pec’ and ‘polspline’ packages to compute the ICI.
